# The interaction between the proliferating macroalga *Asparagopsis taxiformis* and the coral *Astroides calycularis* induces changes in microbiome and metabolomic fingerprints

**DOI:** 10.1038/srep42625

**Published:** 2017-02-20

**Authors:** Stéphane Greff, Tânia Aires, Ester A. Serrão, Aschwin H. Engelen, Olivier P. Thomas, Thierry Pérez

**Affiliations:** 1 Institut Méditerranéen de Biodiversité et d’Ecologie marine et continentale, IMBE UMR 7263 CNRS/IRD/Aix Marseille Université/Avignon Université. Station Marine d’Endoume, rue de la Batterie des Lions, 13007 Marseille, France; 2 CCMAR–CIMAR Centre for Marine Sciences, University of Algarve, Campus de Gambelas, 8005–139 Faro, Portugal; 3 GEOAZUR, UMR 7329 CNRS/Université de Nice Sophia Antipolis, Faculté des Sciences, Parc Valrose 06108, Nice, France; 4 Marine Biodiscovery, School of Chemistry, National University of Ireland Galway, University Road, Galway, Ireland

## Abstract

Mediterranean Sea ecosystems are considered as hotspots of biological introductions, exposed to possible negative effects of non–indigenous species. In such temperate marine ecosystems, macroalgae may be dominant, with a great percentage of their diversity represented by introduced species. Their interaction with temperate indigenous benthic organisms have been poorly investigated. To provide new insights, we performed an experimental study on the interaction between the introduced proliferative red alga *Asparagopsis taxiformis* and the indigenous Mediterranean coral *Astroides calycularis*. The biological response measurements included meta–barcoding of the associated microbial communities and metabolomic fingerprinting of both species. Significant changes were detected among both associated microbial communities, the interspecific differences decreasing with stronger host interaction. No short term effects of the macroalga on the coral health, neither on its polyp activity or its metabolism, were detected. In contrast, the contact interaction with the coral induced a change in the macroalgal metabolomic fingerprint with a significant increase of its bioactivity against the marine bacteria *Aliivibrio fischeri*. This induction was related to the expression of bioactive metabolites located on the macroalgal surface, a phenomenon which might represent an immediate defensive response of the macroalga or an allelopathic offense against coral.

Macroalga–coral interactions received a great attention during the last decades in tropical marine ecosystems, leading to the general assumption that coral reef degradation is at least partly linked to macroalgal proliferation^1^,^2^. Phase shifts from coral to macroalgal dominance, factors that trigger coral diseases (*e.g*. Black or White Band) and declines at regional scales are continuously under investigation in the tropics. Impacts of contaminants, sea temperature increase, and ocean acidification are among the most common factors responsible for the degradation of coral reefs^3^. Many other factors or actors may affect coral resilience and therefore could play a central role in the ecological balance of these ecosystems. Macroalgal overgrowth may represent physical barriers to larval settlement, acting as abrasive or shading factors, but also as a source of bioactive metabolites or pathogens that could affect coral health^4^. These processes of competition are mainly studied in tropical rather than in temperate ecosystems^5^,^6^,^7^.

The Mediterranean Sea is a hotspot of biodiversity but also of biological introductions. Up to now, 128 introduced macroalgal species have been reported in the Mediterranean Sea, which represent more than 10% of the known marine flora^8^. These introductions of non–native species represent a major threat to indigenous corals as well as ecosystem structure and functioning (for a review see refs 9 and 10). For instance, macroalgae of the genus *Asparagopsis* spp. are considered invasive in the Mediterranean Sea because of their seasonal predominance in some local ecosystems, overgrowing up to 100% of the substratum surface^11^,^12^. This genus is composed of a complex of cryptic species^13^, but only two species are presently accepted, *Asparagopsis taxiformis* (Delile) Trevisan de Saint–Léon and *A. armata* (Harvey), both having a cosmopolitan distribution and being well established in the Mediterranean Sea. However, the presence of *A. taxiformis* in the Southwestern Mediterranean basin is quite recent, as it was observed for the first time in 1999 along the coast of Morocco^11^,^14^. It is currently suspected to spread rapidly^11^,^15^. Despite the absence of massive building corals in the Mediterranean Sea, Scleractinians are present and shape some ecosystems locally. This is especially the case for the orange coral, *Astroides calycularis* (Pallas, 1766) (Scleractinian, Dendrophillidae), an azooxanthellate colonial coral, listed as a threatened species, which represents a dominant component of the coralligenous in some places of the South Western basin of the Mediterranean Sea^16^. Co–existence of *A. calycularis* and *A. taxiformis* can be observed in the Alboran Sea and in various places off the coasts of Algeria (Fig. 1). We therefore decided to implement a case study on the interaction between these two species in a temperate region.

Our study is divided in two complementary approaches combining meta–barcoding to detect putative shift in the composition of the associated microbiome and untargeted metabolomics to measure biological responses of both holobionts. We wanted also to assess the short and longer term effects of the interaction, physical processes, chemical releases and pathogenic transfers. First, an aquarium experiment was performed to study the short term effects of the interaction (15 days) between the macroalga on the coral health through the inspection of their behavioral and metabolic responses, but also through the description of the associated bacterial community. In a second set of experiments dedicated to the study of the longer term interaction, the metabolomic fingerprints were obtained from coral and macroalga specimens sampled in the Alboran Sea, from an area where *A. taxiformis* is well–established and outcompeting *A. calycularis* for several years (personal observations).

## Results

### Aquarium experiment

**Figure 2:**
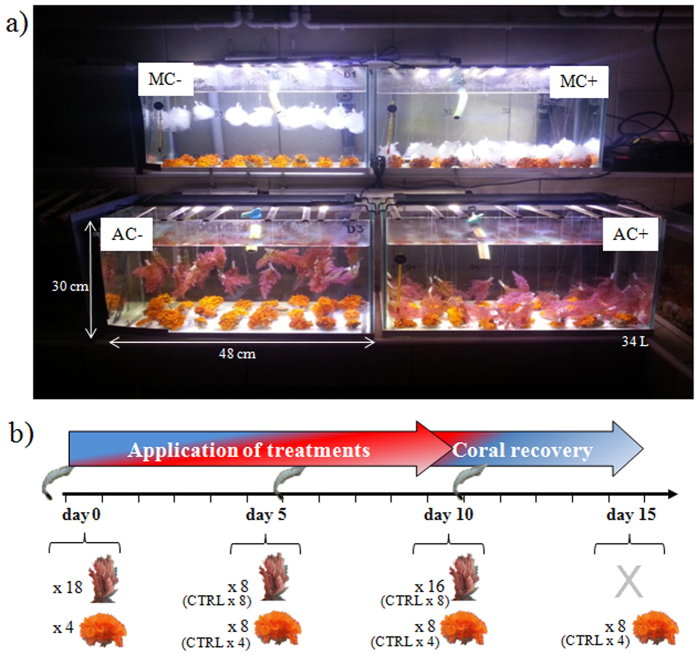
(**a**) Experimental aquaria showing treatment design. (**b**) Experimental scheme with sampling dates and numbers of sampled individuals according to treatments and control (CTRL). MC− corals in contactless interaction with mimic macroalgae; MC+ coral in contact interaction with mimic macroalgae; AC− corals in contactless interaction with macroalgae; AC+ corals in contact interaction with macroalgae. Shrimps figure coral indicates feeding dates (images by S. Greff).

Short term effects of the interaction between the temperate coral *Astroides calycularis* and the red macroalga *Asparagopsis taxiformis* were investigated with an aquarium experiment (Fig. 2). Treatment consisted of control coral and control macroalga without interaction, contact interaction between coral and macroalga (AC+) and contactless interaction (species together but without touching each other, AC−). In order to test if the interaction effect on coral could only be mechanical, additional treatments were set up with artificial mimics macroalgae replacing natural ones either in contact (MC+) and contactless (MC−) interaction with corals. Coral polyp activity, coral and macroalgal microbiomes as well as metabolomic fingerprints and macroalgal bioactivities were studied during 15 days: 10 days during the application of treatments (interactions) followed by 5 days of coral recovery.

#### Seawater composition and temperature

Phosphate, nitrate and nitrite were monitored every 3 days during the experiment (see Supplementary Figure S1). Phosphate concentrations had an average value of 0.10 ± 0.02 *μ*M (±SE) for all treatments and dates reaching a maximal value of 0.41 and 0.46 *μ*M at day 12 and 15 respectively. Nitrate concentrations ranged from 1.4 to 9.9 *μ*M (mean of 4.8 ± 0.3), while nitrite concentrations reached a maximal value of 0.14 *μ*M at day 9 (mean of 0.06 ± 0.01). Ion concentrations for the Northwestern Mediterranean Sea generally range from 0.03 to 0.14 *μ*M for phosphate and from 0.16 to 2.68 *μ*M for nitrate^17^, while the level of nitrites is generally very low (<0.1 *μ*M)^18^. Overall, the mean concentrations of all inorganic ions remained rather constant among aquaria, except for phosphates that reached a maximal concentration in the treatment containing corals in contactless interaction with macroalgae at day 12 and 15, and contact aquarium at day 12, so when the interaction was finished. Nitrate and phosphate concentrations in the aquaria were higher but in the same order of magnitude with those in the sea^17^,^18^. Recorded water temperatures ranged from 15.8 to 17.7 °C with a mean value of 16.5 ± 0.4 °C (±SD, Supplementary Figure S2). The range of these variations of abiotic factors shows that the interaction experiments were conducted as close to natural conditions as possible.

#### Coral behavior

**Figure 3:**
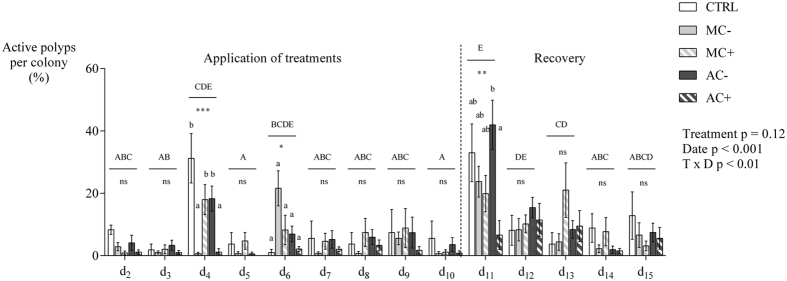
Daily mean percentage of active polyps per coral colony under each of four treatments. CTRL corals alone for controls, MC− corals in contactless interaction with mimic macroalgae, MC+ corals in contact interaction with mimic macroalgae, AC− corals in contactless interaction with macroalgae, AC+ corals in contact interaction with macroalgae. Mean ± SE (n = 7 for treatments, n = 4 for controls). Non–parametric permutation test (1e^5^) with repeated measures (treatment: p = 0.12, date: p < 0.001, treatment × date: p = 0.002). Friedman’s test followed by Nemenyi’s multiple comparison post hoc test were used to compare coral activity between dates (Q = 140.4, p < 0.001). Uppercase letters indicate different coral activities between dates (whatever the treatment as coral activities were pooled for this factor for each date). Kruskal–Wallis test followed by Steel–Dwass–Critchlow–Fligner post hoc test were used to test treatment differences on coral activity for each date. *p < 0.05, **p < 0.01, ***p < 0.001, ns figure non significative. Lowercase letters indicate significantly different groups according to treatments (p < 0.05). Corals were fed with crushed shrimps at the end of day 5 and day 10.

The polyp activity was daily surveyed on the same 7 tagged colonies per interaction treatments (only 4 individuals for control) and expressed as the percentage of active polyps per colony. Polyp activity varied significantly in time, but no clear pattern could be related to any treatments (treatment p = 0.12, date p < 0.001, date × treatment p = 0.002, non–parametric permutation test analysis with repeated measures using 1e^5^ permutations). Temporal variation in polyp activity seems to be mostly associated to the day of feeding (end of day 5 and 10, Fig. 3). At day 4, corals in contactless interaction with mimic macroalgae and contact interaction with macroalgae differed from the control with few active polyps (Kruskal–Wallis, K = 23.2, p < 0.001, SDCF post hoc test). At day 6, no differences between treatments were recorded although a higher activity of polyps was recorded that may be attributed to coral feeding at the end of day 5 (Friedman’s test, Q = 140.4, Nemenyi’s post hoc). The same effect of the feeding was recorded at day 11, with up to 42% of active polyps in contactless interaction with macroalgae. At this date, corals in contactless interaction with macroalgae showed the highest activity while coral in contact interaction with macroalgae showed the lowest one, with intermediate values for coral controls.

#### Associated microbial communities

**Figure 4:**
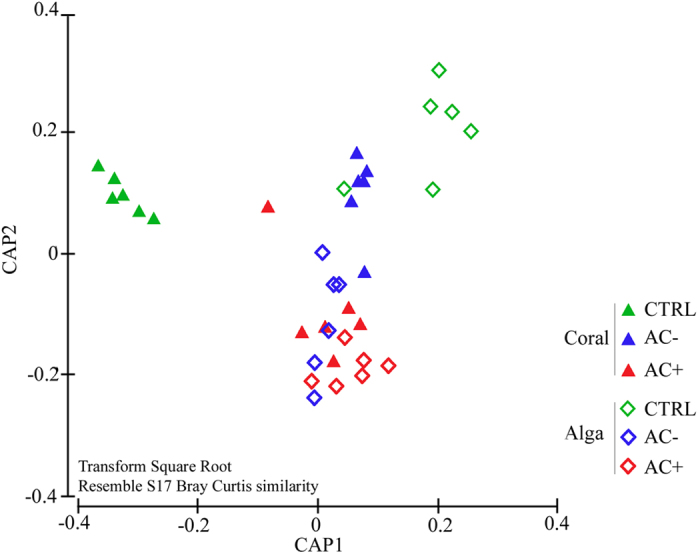
Canonical Analysis of Principle coordinates (CAP) plot of bacterial communities associated to the coral *Astroides calycularis*. Both species under control conditions (CTRL), with contactless interaction (AC−) or contact interaction (AC+) at day 5 and 10 for macroalgae, at day 5 and 15 for corals, pooled according to this factor. Canonical analysis was performed on OTU composition and abundances that were square root transformed and distance matrix was calculated using Bray Curtis similarities.

Microbiomes, in this case associated bacteria and Archaea, were studied at day 5 and 10 for the macroalga and at day 5 and 15 for the coral in order to assess changes in both associated communities according to the type of interaction (no interaction, contactless and contact interactions) and duration. For a given type of interaction, no significant difference in composition of the microbial communities could be detected over time (PERMANOVA p = 0.133). A total of 54,696 unique operational taxonomic units (OTUs) were detected in the entire dataset of 1,564,936 sequences. The total number of OTUs, Chao1 richness, evenness and Shannon index were 10–16% higher in *A. taxiformis* than in *A. calycularis*. Since bacterial composition did not change with time, comparisons between treatments were achieved on time–pooled samples. The total number of OTUs and OTU richness increased from control treatments to contactless interaction and finally contact interaction by 18% (ANOVA p = 0.042, Tukey test p = 0.033). Macroalga and coral contained globally distinct bacterial communities (PERMANOVA p = 0.001, PERMDISP p = 0.089; Fig. 4), although both were dominated by the same bacterial groups including the genera *Rhodobacteraceae, Flavobacteraceae, Propionibacteraceae* and *Alteromonadaceae*. Contributions of individual OTUs to the interspecific difference between macroalga and coral microbiome were smaller than 0.24%. Eighty low abundance OTUs were unique for *A. calycularis*, whereas 50 low abundance OTUs were unique for *A. taxiformis* and 234 OTUs associated with the macroalga but not with the coral.

**Figure 5:**
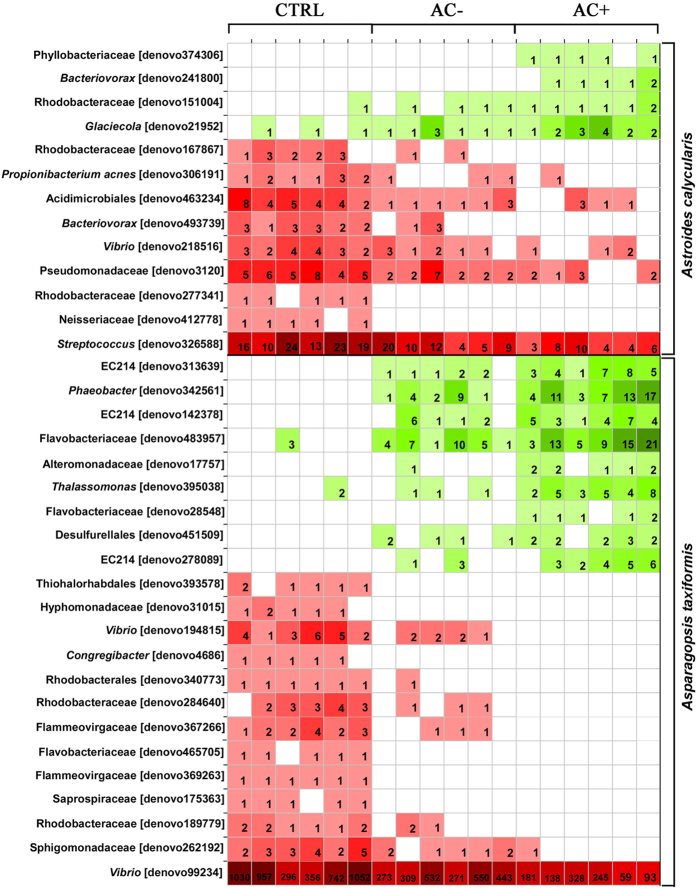
Heatmap summarizing the abundance of OTUs associated with the macroalga *Asparagopsis taxiformis* or the coral *Astroides calycularis* with correlation coefficient > 0.7000 based on 16 S rRNA gene bacterial profiles under experimental conditions: CTRL macroalgae/corals alone for control; AC−macroalgae/corals in contactless interaction with corals; AC+ macroalgae in contact interaction with corals.

Overall, interactions induced changes in both species microbiome (PERMANOVA p = 0.025, PERMDISP p = 0.197), but only control *versus* contact interactions showed significant differences in the pairwise post hoc tests (p = 0.009). Canonical analysis of principle components showed the clear separation of community composition between species, especially well pronounced under control conditions, but these differences faded as the interactions intensified (Fig. 4). The abundance of some low abundance OTUs showed correlations (coefficient >0.7000) with treatments for *A. taxiformis* and *A. calycularis* (Fig. 5).

Coral associated communities contained four OTUs which increased in abundance with increasing interaction (positive correlation) and nine OTUs decreased in abundance with increasing interaction (negative correlation) with *A. taxiformis*. All coral associated OTUs increasing with interaction intensity were *Proteobacteria* of the phyla *Phyllobacteriaceae, Bacteriovoracaceae* (genus *Bacteriovorax*), *Rhodobacteraceae*, and *Alteromonadaceae* (genus *Glaciecola*). The negative correlating OTUs were also mainly *Proteobacteria* (phyla *Rhodobacteraceae, Vibrionaceae, Pseudomonadaceae, Rhodobacteraceae, Neisseriaceae* and *Bacteriovoraceae*) as well as two *Actinobacteria* (orders *Actinobacteria* and *Acidimicrobiia*) and *Firmicutes* (genus *Streptococcus*).

Nine low abundance OTUs associated to *A. taxiformis* correlated positively with coral interaction (Fig. 5). These OTUs were mostly represented by *Proteobacteria* (orders *Rhodobacterales, Alteromonodales* (2x) and *Desulfurellales*), three OTUs of the phylum *SBR1093* from class *EC2214* and *Bacteriodetes* of the phylum *Flavobacteriaceae*. Negative correlations with coral contact were observed with 13 OTUs consisting of nine *Proteobacteria* (orders *Thiohalorhabdales, Rhodobacteriales* (4x), *Vibrionales* (2x), *Alteromonodales, Sphingomonadales*) and four *Bacteroidetes* (phyla *Flammeovirgaceae* (2x), *Saprospiraceae* and *Flavobacteriaceae*; Fig. 5). All of these were also low abundance OTUs with the exception of one of the *Vibrionales* OTUs (family *Vibrionaceae*), which was rather abundant especially on *A. taxiformis* under control conditions (read average 739 ± 39, ±SD).

#### Coral metabolomic fingerprints

A mixture of MeOH:DCM (1:1) was used to extract coral metabolome, that was then fractioned with H_2_O, MeOH and DCM. Aqueous fractions were not analyzed as they contain high concentrations of salts that are inconsistent with the electrospray ion source. Only methanolic fractions were analyzed as they contained the major mass of the extract (10 ± 2 mg, mean ± SD) compared to DCM fractions (3 ± 1 mg). UHPLC–QqToF analyses of the coral methanolic fractions were acquired exclusively in positive mode (2289 ions selected) as the species was known to produce easily ionisable nitrogenated metabolites^19^,^20^,^21^. Acquisition in negative mode was achieved but resulted in only a weak detection of compounds compared to positive mode (22 metabolites). All previously known metabolites of *A. calycularis*^19^,^20^,^21^, except 2–amino–6–(1R,2S–dihydroxypropyl)–3–methylpterin–4–one were detected. Based on metabolic consistency considerations and the *m*/*z* values of the ions detected in the chromatograms, the list of known metabolites was expanded with a number of isomers and putative new compounds (see Supplementary Table S1). At *t*_R_ 0.56 and 0.87 min (peaks 2 and 3), two ions (*m*/*z* 387.1775, [C_18_H_23_N_6_O_4_]^+^) are likely to correspond to dihydrogenated orthidines or orthidine E derivatives not previously described for the species. Moreover, orthidine A (*m*/*z* 385.1619, [C_18_H_21_N_6_O_4_]^+^, peaks 5 to 8, retention time between 4.39 and 4.94 min) was added to the list of the three already known orthidines B, C and D. At *t*_R_ 7.14 min (peak 17), an ion with *m*/*z* 325.1654 may reasonably be assigned to *N*–methyl–*N*–propionylaplysinopsin ([C_18_H_21_N_4_O_2_]^+^) completing the five aplysinopin derivatives identified in *A. calycularis*^19^,^21^.

No intraspecific variation of the coral metabolomic fingerprints attributable to experimental treatments or sampling dates was detected (PERMANOVA p = 0.401) (Supplementary Figure S3).

#### Macroalgal metabolomic fingerprints and bioactivities

According to the same procedure employed for coral, MeOH:DCM (1:1) was used to extract macroalgal metabolome, that was then divided into 3 fractions. Aqueous extracts were not analyzed because of high concentrations of salts. Only methanolic fractions were analyzed as they contained the major mass of the extract (18 ± 4 mg, mean ± SD) compared to DCM fractions (1.5 ± 0.4 mg). Metabolomic fingerprints of the macroalgal methanolic fractions were acquired in UHPLC–QqToF in positive (4051 ions selected) and negative (2507 ions selected) modes, together with their bioactivities (gamma) using the Microtox^®^ assay^22^. Positive and negative ions were grouped in the same matrix to reveal the maximal variance in chemical composition.

**Figure 6:**
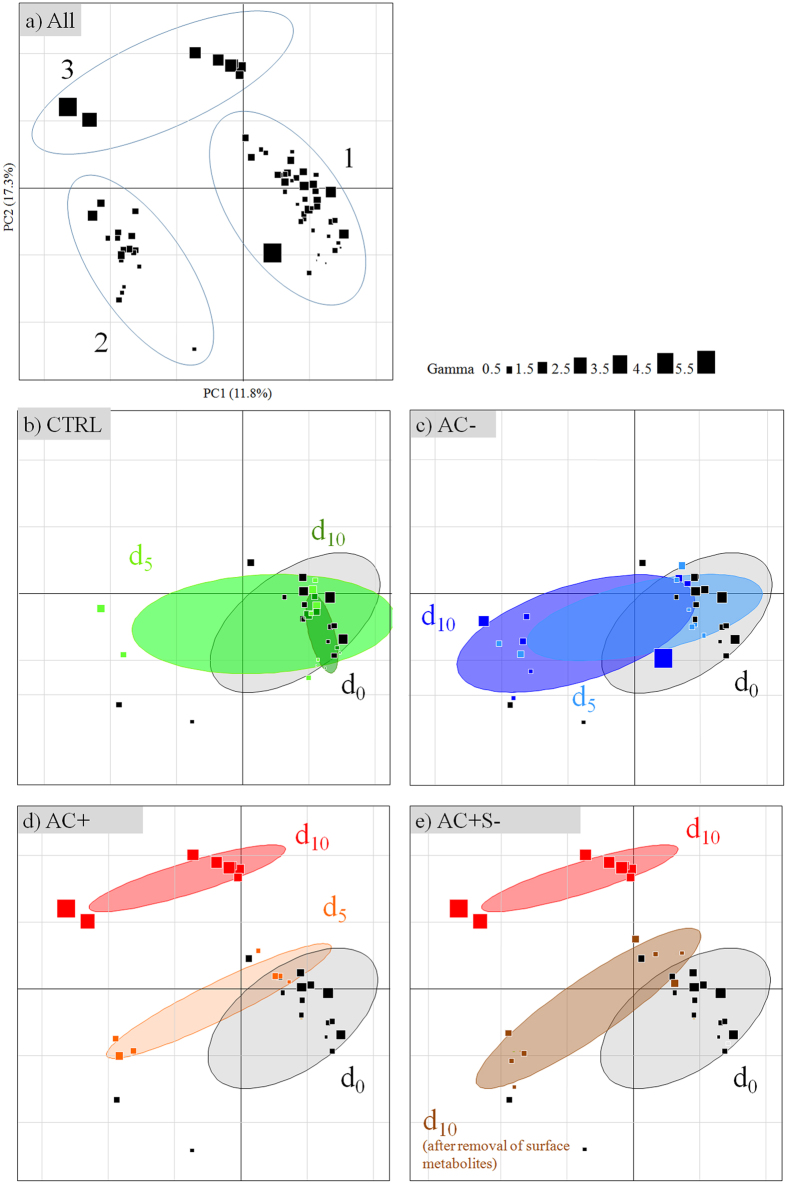
Principal Component Analysis (PCA) of *Asparagopsis taxiformis* metabolomic fingerprints according to experimental conditions (methanolic extracts analyzed in (+)–ESI and (−)–ESI). (**a**) all samples and definition of the three main metabolic groups, (**b**) CTRL macroalgae alone for control, (**c**) AC− macroalgae in contactless interaction with corals, (**d**) AC+ macroalgae in contact interaction with corals, (**e**) AC + S− macroalgae in contact interaction with corals treated for the removal of surface metabolites. d0 = experimental start, d5 and d10 = after 5 and 10 days of treatments. Symbol sizes indicate the bioactivity of extract given in gamma (increasing bioactivities from 0.5 to 5.5). Ellipses are graphical representations of groups without any statistical support.

**Figure 7:**
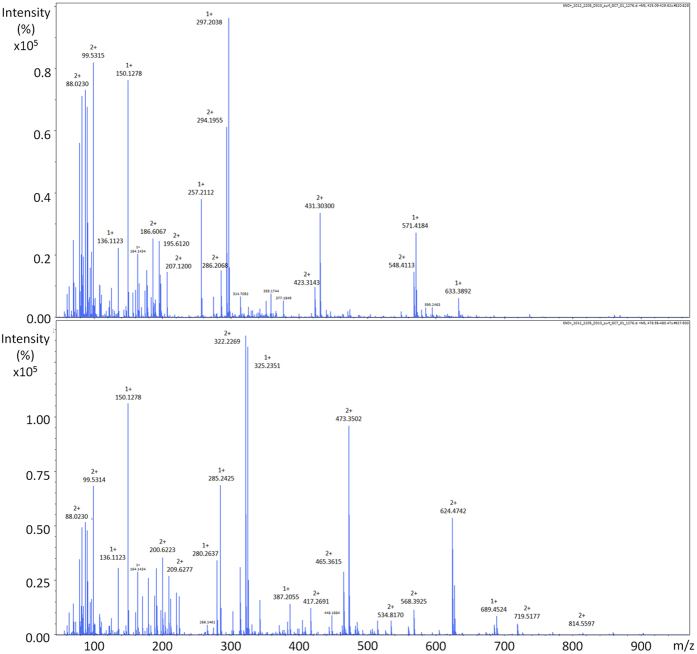
(+)-ESI–mass spectra of two major macroalgal metabolites induced on macroalgal surface after 10 days of contact interaction with corals.

Overall, macroalgal individuals in interaction with the corals exhibited variations both in terms of metabolomic fingerprints and bioactivities. The Principal Component Analysis (PCA) revealed three groups of individuals. Group 1 and 2 represent metabolomic phenotypes which showed the lowest bioactivities, while group 3 the highest (Fig. 6a, all treatments) (Kruskal–Wallis, K = 24.0, p < 0.001 for bioactivities). All selected ions ([M + H]^+^, [M − H]^−^ and potential adducts) potentially responsible for the differences in the chemical composition between macroalgal group 1 and 2 are listed in Table S2. Since many fragmentations occurred in positive mode, only base peak chromatograms were considered. Group 2 metabolomic phenotypes are mostly characterized by metabolites that contain generally 16 to 18 carbons with one nitrogen and 1 to 2 oxygen atoms. On the other hand, negative ions contain 9 to 11 carbons with one nitrogen and 2 to 3 oxygen atoms. The main features of the group 3 metabolomic phenotypes consisted of two major metabolites that likely belong to the same chemical class, according to their mass spectra, their isotopic and fragmentation patterns class (Fig. 7).

**Figure 8:**
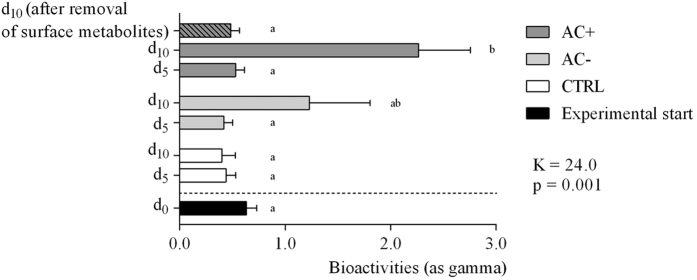
Macroalgal bioactivities (methanol extracts) according to experimental conditions. CTRL macroalgae alone for control; AC− macroalgae in contactless interaction with corals, AC+ macroalgae in contact interaction with corals at different sampling dates (day 0 at experimental start, day 5, and day 10). A subset of macroalgae in contact interaction that were treated to remove surface metabolites are also figured (dashed bar). Mean ± SE (n = 8, except at day 0: n = 18). Kruskal–Wallis test followed by Steel–Dwass–Critchlow–Fligner post–hoc test (K = 24.0, p = 0.001). Letters indicate significantly different groups (p < 0.05).

After 10 days of treatment, the control macroalgae did not show any change in their metabolomic fingerprints (Fig. 6b) nor in their bioactivities (Kruskal–Wallis, p > 0.05; Fig. 8). All control samples mainly belong to Group 1 in the PCA.

Macroalgae placed in contactless interaction exhibited a change in their fingerprints at days 5 and 10 (from group 1 to 2, Fig. 6c) without any change of bioactivity (gamma = 0.4 ± 0.1 and 1.2 ± 0.6 at day 5 and 10 respectively, SDCF post–hoc test, p = 0.91 and 0.94 respectively; Fig. 8).

Macroalgae in contact interaction with corals (Fig. 6d) exhibited at day 5, a change in their fingerprints equivalent to macroalgae in contactless interaction with corals, and in the same way without any change of bioactivity (gamma = 0.7 ± 0.1, SDCF post–hoc test, p = 0.99; Fig. 8). On the other hand, macroalgal metabolomic fingerprints were deeply modified at day 10 (to group 3 on PCA) and a significant increase of bioactivity was observed (gamma = 2.3 ± 0.5, SDCF post–hoc test, p = 0.005; Fig. 8).

#### Effect of the lipophilic extraction of the macroalgal surfaces

In order to determine the location of the day–10–induced metabolites, eight macroalgae in contact interaction with corals were quickly soaked in 10 mL cyclohexane to extract lipophilic surface metabolites (Fig. 6e). Metabolomic fingerprints of the macroalgae after extraction became similar to those at day 5 with a corresponding decrease of bioactivity (0.5 ± 0.1, Fig. 8). So there is a high probability that these two biomarkers contributed to the most of the macroalgal bioactivity and are located at its surface. The non–polarity of these metabolites was evidenced by their solubility in cyclohexane.

### *In situ* observations

**Figure 9:**
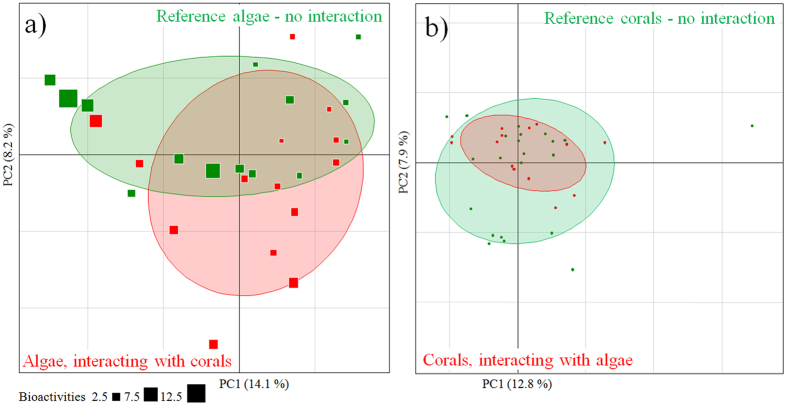
Principal Component Analysis (PCA) of (**a**) *Asparagopsis taxiformis* metabolomic fingerprints of macroalgae sampled *in situ* in contact interaction with the coral *Astroides calycularis* and references (no interaction with corals) (methanolic extracts analyzed in (+)–ESI and (−)–ESI). (**b**) *Astroides calycularis* metabolomic fingerprints of coral sampled *in situ* in contact interaction with the macroalgae *Asparagopsis taxiformis* and references (no interaction with macroalgae) (methanolic extracts analyzed in (+)–ESI) (scores plot). Bioactivities of macroalgal extracts are given as gamma. Ellipses are graphical representations of groups without any statistical support.

Because the experiments in aquaria could not last more than 15 days (due to the difficulty of keeping macroalgae in these conditions), we decided to assess the long term impact of this interaction *in situ*. An assessment of *in situ* interaction effects was made using the metabolomic approach combining negative (1316 ions selected) and positive (2621 ions selected) modes. Data on both species in contact interaction were compared to data obtained from specimens without any visible interaction, the latter being defined as references. Metabolomic fingerprints of both species did not show differences between references and closely interacting specimens (separate PERMANOVA per species: p = 0.08 for macroalgae, p = 0.45 for corals; Fig. 9). Macroalgal bioactivities of field material exceeded and varied more than those measured from macroalgae in the *ex situ* experiment (gamma = 0.8–13.2), likely due to the influence of a higher number of factors such as herbivory and competition with other sessile organisms in natural conditions. Reference macroalgae without interaction with corals exhibited a higher mean bioactivity of 3.7 ± 0.9 (mean ± SE) than those interacting with corals (2.4 ± 0.4), but this difference was not supported by statistics (Mann–Whitney test, p = 0.635).

## Discussion

The overall decline of coral reefs in tropical seas facing global change and their replacement by macroalgae in many reefs have been a source of motivation to study biotic interactions between corals and macroalgae (see refs 4 and 23 for a general review on coral–macroalgal competition). The various processes by which corals and macroalgae interact can be summarized as follows: (i) overgrowth or smothering, depriving the outcompeting species of resources (space, light, nutrients); (ii) shading and overtopping; (iii) physical barrier for recruits; (iv) direct contacts leading to physical damages such as abrasion; (v) chemical mediation^4^,^23^. In temperate regions, coral–macroalga interactions also take place but they remain poorly investigated^5^,^6^,^7^,^24^,^25^. While coral–macroalga competition studies focused mostly on physical mechanisms^4^, it is nowadays well accepted that chemical and biological factors may play an important role in the competition. Indeed metabolic resources are required to defend a macroorganism against predators or competitors, and its associated microbial biofilm can provide resources as well as a first line defense^26^,^27^.

Various studies already demonstrated the adverse effects of macroalgal overgrowth on the settlement, growth and survival of temperate corals such as *Balanophyllia elegans*^6^, and *Oculina arbuscula*^24^,^25^. For instance, physical contacts of *Stephanocystis osmundacea* and *Dictyota binghamiae* with *B. elegans* lead to polyp retraction, resulting in increasing coral vulnerability^6^. Physical effects have been confirmed with the use of mimic plastic macroalgae that exhibited the same adverse effects as natural macroalgae. In our study, the behavioral response of the coral appeared not to be affected by the physical contact with *A. taxiformis*. We wanted to verify if contacts with mimic/natural macroalgae could represent a discomfort for feeding. We did not observe any behavioral disturbance during our short term experiment conducted in aquaria. This might be explained by the aquaria low hydrodynamics, which limited abrasion processes that were observed *in situ*^6^. As macroalgae might affect corals at another biological level of organization, we assessed the effects of the interaction on the coral microbiome and metabolome in *ex situ* experiment.

As every organism is associated with microbes, it is becoming increasingly obvious that these associations can greatly influence host health and its metabolism^28^,^29^,^30^. Our results add to the evidence of species specificity of host associated bacterial communities, when no interaction occurs, as previously demonstrated in several studies on corals and macroalgae^31^,^32^,^33^,^34^,^35^. These associated communities can be involved in the protection of the host from pathogen intrusion and fouling for example by the production of bioactive compounds^26^,^27^. However, our experimental study demonstrated that these associations are not static, as indicated by the significant changes from species specific to intermediate and a new state of community when going from two isolated holobionts to an indirect, and to a direct contact between the macroalga and coral. Thus, the interspecific differences of bacterial communities disappeared with increasing intensity of the interaction. In the applied experimental setup, contactless interaction might be equivalent to a distance interaction probably involving a mix of transmitted bacteria and compounds between macroalga and coral, which cannot be disentangled in the current study, but have been elegantly demonstrated for indirect effects of macroalgae on corals^36^.

Analyses at the OTU level showed some increasing (positive correlation) or decreasing (negative correlation) abundances as the intensity of interactions increase. For both hosts, OTUs associated to negative correlations with interaction intensity were more common than positive ones, indicating that more OTUs are negatively affected by the interaction strength, rather than benefiting from the interaction. Many OTUs represented bacterial groups present in both positive and negative correlations, indicating that, based on 16 S rRNA, closely related OTUs differ in their characteristics. For more details and a better understanding of the functional/metabolic role of these specific OTUs, bacterial isolation and functional characterization (metagenomics) of these OTUs is needed. Bacterial OTUs that increased abundance with the intensity of interaction are not those previously related to coral diseases, as in the case of *Vibrio* sp., *Arcobacter* sp., *Aeromonas* sp., *Cyanobacterium* sp., *Clostridium* sp., *Pseudoalteromonas* sp. and *Shewanella* sp.^37^,^38^,^39^,^40^. Some of these bacteria, like *Vibrio* sp. were actually quite abundant on *A. taxiformis* under control conditions (without the presence of coral), but decreased in abundance as the intensity of interaction increased. Bacteria associated with seaweed diseases as overviewed by Egan *et al*.^41^ provided a better match with the correlating OTUs with the intensity of interaction.

Setting up our experiments, the objectives were also to discriminate between physical and chemical factors important in the coral–macroalga interaction. The contribution of chemical factors may be particularly significant in non–native species, as they may harbor new arsenal of metabolites^42^. In the Western Mediterranean Sea, the introduced species *Lophocladia lallemandii* and invasive *Caulerpa cylindracea* interacting with *Cladocora caespitosa* during a 6–year–survey did not trigger coral necrosis, despite the fact that macroalgae were steadily overgrowing corals^7^. Moreover, bioactivities of coral extracts measured by the Microtox^®^ assay did not show any differences between exposed and non–exposed *Cladocora*, suggesting the absence of strong changes at the metabolomic level. Conversely, the invasive *Caulerpa cylindracea* and *Womersleyella setacea* (Rhodophyta) affected the survival and growth of the sea–fan *Paramuricea clavata* with increasing necrosis rates^5^. This showed that effects on native ecosystems can be highly variable among species and according to the exogenous species. The main triggering process also strongly depends on the influence of local environmental factors (*i.e*. disturbances, herbivore pressure, nutrient intakes)^4^ and McCook *et al*.^4^ pointed out the difficulties to disentangle correlative factors during empirical studies.

When in contact interaction with the coral *A. calycularis, A. taxiformis* exhibited a propensity to trigger the biosynthesis of metabolites and in the same time increasing its bioactivity. The production of lipophilic bioactive metabolites can be a real competitive tool fostering the establishment in an indigenous community^43^. Interestingly, using the same test to measure bioactivity, Martí *et al*.^44^ previously revealed that *Asparagopsis armata*, in its tetrasporophyte stage, were highly bioactive compared to other macroalgae of the Northwestern Mediterranean Sea. *Asparagopsis armata* was already known to synthesize halogenated compounds active against epiphytic microbes (*e.g*. bromoform or dibromoacetic acid)^45^ or herbivores (*e.g*. bromoform)^46^,^47^, but no study could evidence the production of chemicals induced by an interaction with sessile invertebrates. Our experimental design allowed detecting a rather fast induction process of toxic chemicals when the macroalga is placed in contact with a coral. The group 2 metabolomic phenotype observed after 5 days of contact with corals, but also when the coral was in contactless interaction after 10 days, is therefore likely to contain biosynthetic precursors leading to the bioactive metabolites characteristic of the group 3 phenotype. Due to their most probable molecular formula and overall chemical homogeneity, the metabolites characteristic of the group 2 may putatively correspond to long chain aldehydes or sphingosine derivatives. Finally, after 10 days of contact interaction, induced bioactive metabolites are concentrated at the macroalgal surface, and this may be viewed equally as a defense response of the macroalga or a macroalgal offense against the coral.

In temperate latitudes where ecosystems are dominated by macroalgae, research has mainly been focused on macroalgae–macroalgae, macroalgae–pathogens or macroalgae–herbivores interactions^48^,^49^,^50^,^51^,^52^. In contrast, we contributed to fill in gaps of knowledge regarding temperate coral–macroalgal interaction using for the first time combined approaches of metabolomics and bacterial meta–barcoding. Trends observed for metabolomics and meta–barcoding analyses are difficult to merge towards a unique mechanism of response during the interaction between organisms. A general trend towards uniformity of the microbial communities of both macroalga and coral was found under interactive conditions, whereas a rather quick shift in metabolomic fingerprint was only detected in the macroalga and not in the coral. Interestingly, *A. taxiformis* is able to induce specific metabolites towards the production of large bioactive molecules. Because this species was recently introduced, further investigations should be conducted to provide insights on how this macroalga may shape and impact temperate native ecosystems.

## Methods

### Biological Material

*Astroides calycularis* (Pallas, 1766) (Scleractinia, Dendrophillidae) is an azooxanthellate coral, which is found in the Strait of Gibraltar, in the Alboran Sea, along the Algerian and Tunisian coasts, in some places of the Tyrrhenian Sea and in the Adriatic Sea^53^. It forms colonies of polygonal polyps growing mainly on vertical walls, both in light or semi–dark conditions, from shallow waters to 50 m depth.

*Asparagopsis taxiformis* (Delile) Trevisan de Saint–Léon (Rhodophyta, Bonnemaisoniaceae) is a cosmopolite macroalga, widely distributed in sub–tropical to tropical waters. At the moment, six different genetic lineages have been evidenced^13^,^54^,^55^, which might represent a complex of cryptic species. In the western Mediterranean Sea, only *A. taxiformis* lineage 2 was recorded^13^,^54^ and is generally growing on rocky substrates between 0 and 30 m depth. Its complex heteromorphic haplo–diplophasic life cycle alternates between diploid sporophytes, tetrasporophytes and gonochoric gametophytes. In this study, we focused on gametophytes only.

### Sampling and experimental designs

#### Aquarium experiment

Seventy colonies of *A. calycularis* were sampled in Ceuta (Spain) (35°54′25.39″N, 5°17′17.46″W) the 28^th^ of April 2014 at 7–10 m depth, and maintained in several seawater tanks until their delivery to the aquaria of the Station Marine d’Endoume (Marseille) where they were acclimated an entire week. *Asparagopsis taxiformis* lineage 2 was sampled the 6^th^ May 2014 in La Ciotat (France) (43° 9′57.26″N, 5°36′32.40″E) and was subsequently used for the implementation of the experiment.

Twenty–four colonies were placed in any of the four 34 L treatment aquaria for an additional week of stabilization under controlled light conditions (LED panel EcoLED 18 W 1080 Lumens, 12 h light/dark alternately). Twelve other coral colonies and macroalgae alone were placed in separate 21 L aquaria for controls under the same conditions of light alternation. All aquaria were placed in open–circuit flow (flow rate = 160 ± 50 mL.min^−1^, mean ± SD) (Fig. 2). By this way, we intentionally limited variations due to environmental factors, and specifically focused on the putative mechanisms underlying coral–macroalgal interaction.

The experiment took place from the 12^th^ of May to the 27^th^ of May 2014 (day 0 to 15). In the two first aquaria, corals were placed with mimic macroalgae (synthetic filter wadding from Riwalon^®^) either in contact (MC+) or contactless interaction (MC−) to simulate physical discomfort/shading to corals. In the third aquarium, coral colonies and macroalgae were put in the same aquarium without any direct contact (AC−, contactless interaction). In the fourth aquarium, coral colonies were placed in contact interaction with macroalgae (AC+; Fig. 2). During the experiment, corals were fed 3 times, the day before the start of the experiment and at the end of day 5 and of day 10, with ground shrimp mixed with seawater.

For the biological effect measurements, both organisms (entire colonies removed for corals) were sampled at the beginning of experiments (day 0) and then every 5 days (day 5, 10 and 15) (n = 8 for treatments, n = 4 for controls). At day 10, eight supplementary macroalgae (0.176 ± 0.098 g DM, mean ± SD) in contact interaction with corals were soaked in 10 mL cyclohexane, swirling them 30 s according to De Nys’ protocol^56^ to recover macroalgal metabolites at the surface. At day 15, only corals were sampled after a recovery phase from day 10 to day 15 (no macroalgae in aquaria).

#### In situ experiment

Samples of co–occurring *A. calycularis* (n = 14) and *A. taxiformis* (n = 14) were taken at 7–10 m depth in La Herradura (Spain) (36°43′25.74″N, 3°44′13.35″W) the 1^st^ May of 2015, in order to analyze their metabolomic fingerprint. Macroalgae (n = 14) and corals (n = 22) without any interspecific contact were also sampled as references. Both species were found on vertical walls exposed to currents. The macroalgal coverage was high and visually estimated up to 80%.

### Seawater composition and temperatures

Phosphate, nitrate and nitrite concentrations were measured on aquaria seawaters sampled every 3 days using a BioMate 3 UV/VIS Spectrophotometer (Thermo Scientific^®^) and following standardized protocols^18^. Water temperature was surveyed during the experiment using a water temperature data logger TidbiT v2 UTBI–001 (HOBO^®^).

### Measurements of biological effects

#### Coral behaviour

Polyp activity was daily surveyed on the same tagged colonies (n = 7 for treatments, n = 4 for controls). Polyps were considered active when at least one tentacle was deployed. Data were expressed in percentage of active polyps per colony.

#### Bacterial community characterization

For both *A. calycularis* and *A. taxiformis*, DNA was extracted from three freeze–dried replicates of each interacting and control group. *Asparagopsis taxiformis* was sampled after 5 and 10 days of contact and contactless interaction. *Astroides calycularis* was sampled after 5 and 15 days, so for the latter sampling after 5 days release of interactions. In total, 36 samples were extracted using the Quick–gDNA kit (Zymo Research™) according to the manufacturer protocol.

The total 16 S rDNA region was amplified using the universal primers 27F and 1494r with the following changes to the original protocol^57^: after an initial denaturation at 95 °C for 2 min, conditions were 35 cycles of denaturation at 95 °C for 20 s, annealing at 55 °C for 20 s and extension at 72 °C for 90 s. The final extension was at 72 °C for 3 min. The 25 *μ*L reaction mixture contained 250 *μ*M dNTPs, 0.6 *μ*M of each primer, 1 × 2 PCR buffer mix, 2 *μ*l of template DNA (with a final concentration of about 10 ng.*μ*L^−1^) and 0.3 *μ*L of Taq polymerase (Advantage^®^ 2 Clontech). PCR products were cleaned using ExoFastAP enzyme following the manufacturer protocol (Thermo Scientific™) and amplified DNA was submitted to Molecular Research (MR DNA), Shallowater, Texas where a nested–PCR was performed prior to sequencing. Modified 8 bp key–tagged primer 799F along with the reverse primer 1193R (fragment ~400 bp), which avoid chloroplast cross amplification^58^, were used and PCR conditions were as follow: 95 °C for 3 min, 10 cycles of 95 °C for 20 s, 50 °C for 30 s, 72 °C for 30 s, and a final elongation of 72 °C for 3 min. Samples were pooled together in equal proportions based on their molecular weight and DNA concentrations and purified using calibrated Ampure XP beads. DNA libraries were prepared by following Illumina TruSeq DNA library preparation protocol and paired–end (2 × 250 bp) sequencing performed at MR DNA (www.mrdnalab.com, Shallowater, TX, USA) on a MiSeq following the manufacturer’s guidelines.

All the diversity analyses were performed using the program QIIME 1.8.0: Quantitative Insights Into Microbial Ecology^59^. Sequences were screened for a minimum read length of 350 bp and less than 2 or more undetermined nucleotides. The filtered dataset, containing only high quality sequences, was submitted to a conservative chimera detection filter using the ChimeraSlayer method^60^. Selected high quality chimera–free sequences were clustered into Operational Taxonomic Units (OTUs) within reads using the UCLUST^61^ with a pairwise identity threshold of 0.97.

Representative sequences for each OTU were picked using the “most–abundant” method and OTU sequence alignment was performed with Pynast^59^. The Ribosomal Database Project (RDP)^62^ classifier was used for taxonomic assignment with a 95% confidence threshold. Sequences with the best match for eukaryotes (*i.e*. chloroplasts and mitochondria) were excluded from the OTU table in downstream analyses as well as rare OTUs (singletons and doubletons). To assign each OTU to the closest matching described taxon, searches were performed against the Greengenes reference database (version 12_10)^63^ with a maximum e–value to record an assignment of 0.001. Singletons were removed before analysis.

#### Metabolomic fingerprinting

Methanol (MeOH), dichloromethane (DCM) and acetonitrile of analytical quality (Chromasolv^®^, gradient grade) were provided by Sigma–Aldrich. Eluent additive (formic acid and ammonium formate, LC–MS Ultra grade) were provided by Fluka.

At each sampling date, macroalgae and coral colonies were flash–frozen into liquid nitrogen and then stored at −80 °C before freeze–drying in order to prevent any metabolism shift (n = 8 for treatments, n = 4 for controls). Dried coral polyps (n = 38 ± 16, mean ± SD) were scratched into a first powder sieved at 710 *μ*m. Both organisms were ground in a mixer mill (Retsch^®^ MM400, 30 Hz during 30 s) to yield a fine powder. One hundred milligrams of each sample was extracted 3 times with 2 mL of MeOH:DCM 1:1 (v/v) in an ultrasonic bath (1 min) at room temperature. The filtrates (PTFE, 0.22 *μ*m, Restek^®^) were pooled and concentrated to dryness, depositing the extract on reversed silica (100 mg, non–end–capped C18 Polygoprep 60–50, Macherey–Nagel^®^). The extracts were then fractionated by SPE (Strata C18–E, 500 mg, 6 mL, Phenomenex^®^), with an elution of H_2_O, MeOH, and DCM (5 mL each) initially cleaned with MeOH (10 mL) and conditioned with H_2_O (10 mL). Aqueous fractions were not analyzed because of high concentrations of salts in this fraction that may alter the chromatographic separation and mass ionization processes by electrospray ion source. Considering our experience with both models^19^,^64^, we believed that analyses of aqueous and DCM fractions, providing rather low weight of chemical extracts, would therefore be limited in their interest. The DCM fractions would allow the observation of mainly highly non polar compounds, likely fatty acids or other lipid compounds which are usually of rather low bioactivity. So we rather focused on the MeOH fraction, known to usually contain high concentrations of bioactive specialized metabolites. After analysis, dried MeOH macroalgal extracts were also used for bioactivity assessment using the Microtox^®^ ecotoxicological assay.

Metabolomic fingerprintings were achieved on an UHPLC–QqToF instrument (Dionex Ultimate 3000 equipped with RS Pump, autosampler and thermostated column compartment and UV diode array, Thermo Scientific^®^) coupled to an accurate mass spectrometer (MS) equipped with an ESI source (Impact II, Bruker Daltonics^®^). Mass spectra were acquired in positive and negative modes for macroalgal extracts, in positive mode only for coral extracts. Chromatographic solvents were composed of A: water with 0.1% formic acid (positive mode), or 10 mM ammonium formate (negative mode), and B: acetonitrile/water (95:5) with the same additives. UHPLC separation was performed on an Acclaim RSLC C18 column (2.1 mm × 100 mm, 2.2 *μ*m, Thermo Scientific^®^) using 2 different programs for macroalgal and coral extracts. The macroalgal program consisted of: 2% B during 1 min (2 min for coral), followed by a linear gradient up to 80% B during 5 min (8 min for coral), then 6 min in isocratic mode (2 min for coral); the analysis was followed by an elution of 100% B during 3 min until a return to initial conditions for column equilibration during 3 min for a total runtime of 18 min for both organisms. The elution rate was set at 0.5 mL.min^−1^ with a constant temperature of 40 °C for the column.

Before their injection, macroalgal and coral extracts were resuspended in 2 mL MeOH (with an extra 1/10 dilution for corals). Samples were injected in random order, 10 *μ*L for macroalgal and 2 *μ*L for coral extracts respectively, alternating with a pooled sample (mix of all samples of each organism) injected every 6 samples to grasp MS shift. MS parameters were set as follow for positive mode (and negative mode): nebulizer gas, N_2_ at 31 psi (51 psi); dry gas, N_2_ at 8 L.min^−1^ (12 L.min^−1^), capillary temperature at 200 °C and voltage at 2500 V (3000 V). Data were acquired from 50 to 1200 amu. The mass spectrometer was systematically calibrated with a formate/acetate solution forming clusters on the studied mass range before analysis of a full set of samples. The same calibration solution was automatically injected before each analysis for internal mass calibration.

Raw analyses were automatically recalibrated using the calibrant portion, before exporting data in netCDF files (centroid mode) using Bruker Compass DataAnalysis 4.3. Sets of converted analysis were consequently processed using XCMS software^65^. Each individual ion of each analysis was finally normalized if necessary (modeling the relationship of the intensity of equivalent ions of pooled samples and injection order) according to the methodology described by van der Kloet *et al*.^66^.

#### Bioactivity assays

The bioactivity of macroalgal extracts was assessed using a standardized Microtox^®^ ecotoxicological assay (Microbics, USA). The assay consists of a measure of the natural bioluminescence of the marine bacteria *Aliivibrio fischeri* and its inhibition when exposed to toxic compounds. Extracts were prepared at an initial concentration of 2 mg.mL^−1^, and consequently diluted in artificial seawater with 2% of acetone to facilitate dissolution. Adequate dilutions were prepared to fit Microtox requirements. Data were expressed as gamma (unitless) as gamma takes into account extraction yields (for calculation details see Greff *et al*.^64^).

### Statistical analyses

Non–parametric permutation analysis to test treatments on coral behavior was realized using package “ez”^67^ (1e^5^ permutations) under R^68^. Non–parametric analysis (Kruskal–Wallis test followed by Steel–Dwass–Critchlow–Fligner post–hoc test, and Friedman test followed by Nemenyi post–hoc test) were used to compare bioactivities and coral behavior for separate factors (Friedman for dates with tagged colonies, and Kruskal–Wallis for treatments) and performed in XLSTAT Version 2015.4.01.20575. Principal Components Analysis (PCA) were realized using package “ade4”^69^, while Permutational Multivariate Analysis of Variance Using Distance Matrices (PERMANOVA) tests were performed with function “adonis” with package “vegan”^70^. Regressions to calculate gamma were performed under Statgraphics Centurion XV Version 15.2.11.

## Additional Information

**How to cite this article**: Greff, S. *et al*. The interaction between the proliferating macroalga *Asparagopsis taxiformis* and the coral *Astroides calycularis* induces changes in microbiome and metabolomic fingerprints. *Sci. Rep.* **7**, 42625; doi: 10.1038/srep42625 (2017).

**Publisher's note:** Springer Nature remains neutral with regard to jurisdictional claims in published maps and institutional affiliations.

## Supplementary Material

Supplementary Information

## Figures and Tables

**Figure 1:**
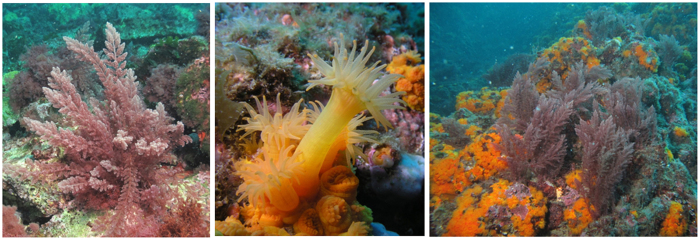
*Asparagopsis taxiformis, Astroides calycularis*, and both organisms in interaction (10 m depth, 25^th^ of April 2013, La Herradura, Spain, images by T. Pérez).
