# Supplementary material for: The interaction between the proliferating macroalga Asparagopsis taxiformis and the coral Astroides calycularis induces changes in microbiome and metabolomic fingerprints
Source: Sci Rep. 2017 Feb 20;7:42625. doi: 10.1038/srep42625 (PMC5317032; doi:10.1038/srep42625)
Supplement: Supplementary Information [file srep42625-s1.pdf]

Supplementary Information

The interaction between the proliferating macroalga *Asparagopsis taxiformis* and the coral *Astroides calycularis* induces changes in microbiome and metabolomic fingerprints

Stéphane Greff[†], *Tânia Aires*[#], *Ester A. Serrão*[#], *Aschwin H. Engelen*[#], *Olivier P. Thomas*[†,⊥,§], *Thierry Perez*[†,*]

[†] Institut Méditerranéen de Biodiversité et d'Ecologie marine et continentale, IMBE UMR 7263 CNRS / IRD / Aix Marseille Université / Avignon Université. Station Marine d'Endoume, rue de la Batterie des Lions, 13007 Marseille, France.

[⊥] GEOAZUR, UMR 7329 CNRS / Université de Nice Sophia Antipolis, Faculté des Sciences, Parc Valrose 06108, Nice, France.

[§] Marine Biodiscovery, School of Chemistry, National University of Ireland Galway. University Road, Galway, Ireland.

[#] CCMAR-CIMAR Centre for Marine Sciences, University of Algarve, Campus de Gambelas, 8005-139 Faro, Portugal.

Figures

Figure S1 – Seawater inorganic ion content (phosphate, nitrate and nitrite in µM) measured every 3 days from day 0 (start of experiment) to day 15 for all aquaria. MC- – corals in contactless interaction with mimic algae, MC+ – corals in contact interaction with mimic algae, AC- – corals in contactless interaction with algae, AC+ – corals in contact interaction with algae, Coral CTRL – corals alone for controls, Algae CTRL, algae alone for controls...3

Figure S2 – Temperature records during experiment in aquarium AC- (corals in contactless interaction with algae). Grey line figures the mean..…4

Figure S3 – Principal Component Analysis (PCA) of coral *Astroides calycularis* metabolomic profiles according to treatments (methanolic extracts analyzed in (+)–ESI). MC- – corals in contactless interaction with mimic algae, MC+ – corals in contact interaction with mimic algae, AC- – corals in contactless interaction with algae, AC+ – corals in contact interaction with algae, CTRL – corals alone for controls, at different sampling dates (experimental start at day 0, day 5, day 10 and day 15) (scores plot). PC1 and PC2 represents 7.2 and 7.8 % of variance for a total of 15.0 %. Ellipses are graphical representations of groups without any statistical support...5

Tables

Table S1 – Coral metabolites determined by UHPLC–(+)–ESIMS (methanolic fraction). RT : Retention time in minutes, BPC Area (%) = base peak chromatograms are given as percentage, mS: mSigma is a constructor quality value of the formula determination. It compares accurate masses and isotopic patterns of experimental formula with theoric ones (value of mSigma closest to zero are representative of maximal fit). Base peak chromatograms were considered for ion formula determination...6

Table S2 – ***Asparagopsis taxiformis* ions responsible for group 2 difference in the Principal Component Analysis (Fig. 6 in main manuscript).** RT: Retention time in minutes, mS: mSigma is a constructor quality value of the formula determination. It compares accurate masses and isotopic patterns of experimental formula with theoric ones (value of mSigma closest to zero are representative of maximal fit). Base peaks chromatograms were considered for ion formula determination.7

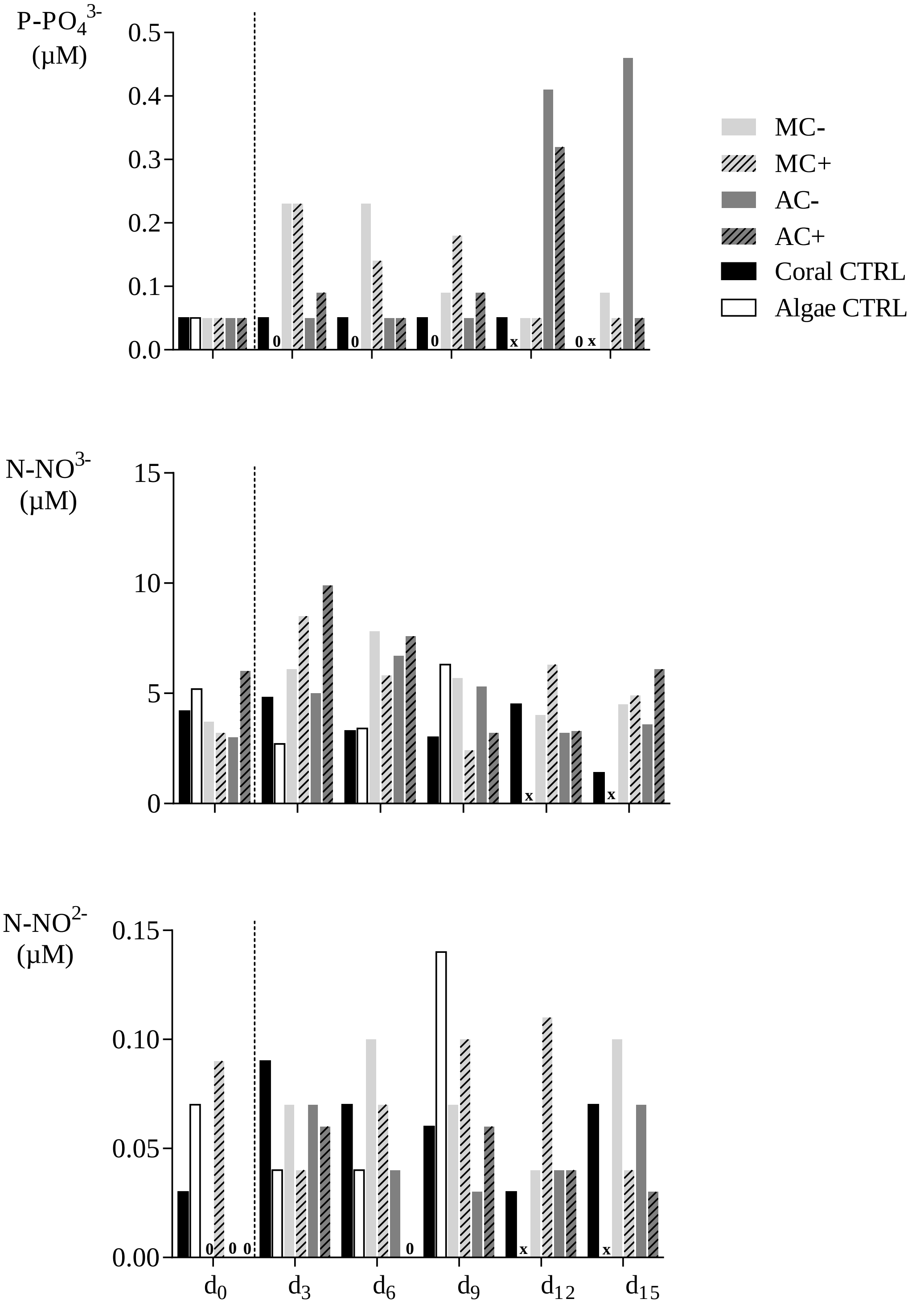

Figure S1 – Seawater inorganic ion content (phosphate, nitrate and nitrite in µM) measured every 3 days from day 0 (start of experiment) to day 15 for all aquaria. MC- – corals in contactless interaction with mimic algae, MC+ – corals in contact interaction with mimic algae, AC- – corals in contactless interaction with algae, AC+ – corals in contact interaction with algae, Coral CTRL – corals alone for controls, Algae CTRL, algae alone for controls.

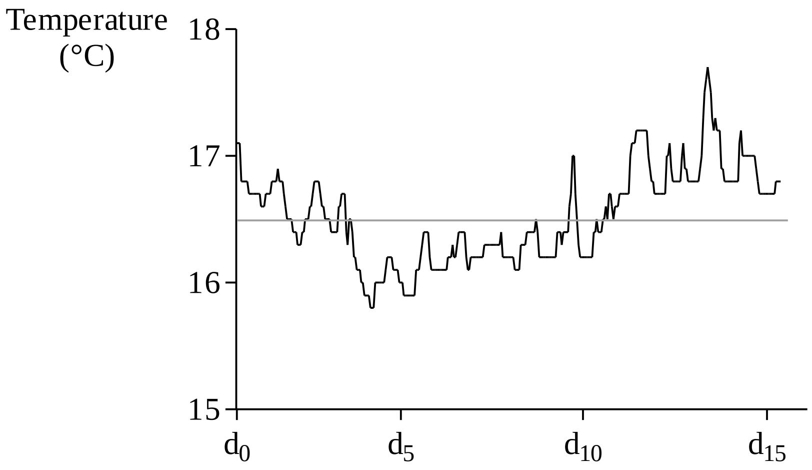

Figure S2 – Temperature records during experiment in aquarium AC- (corals in contactless interaction with algae). Grey line figures the mean.

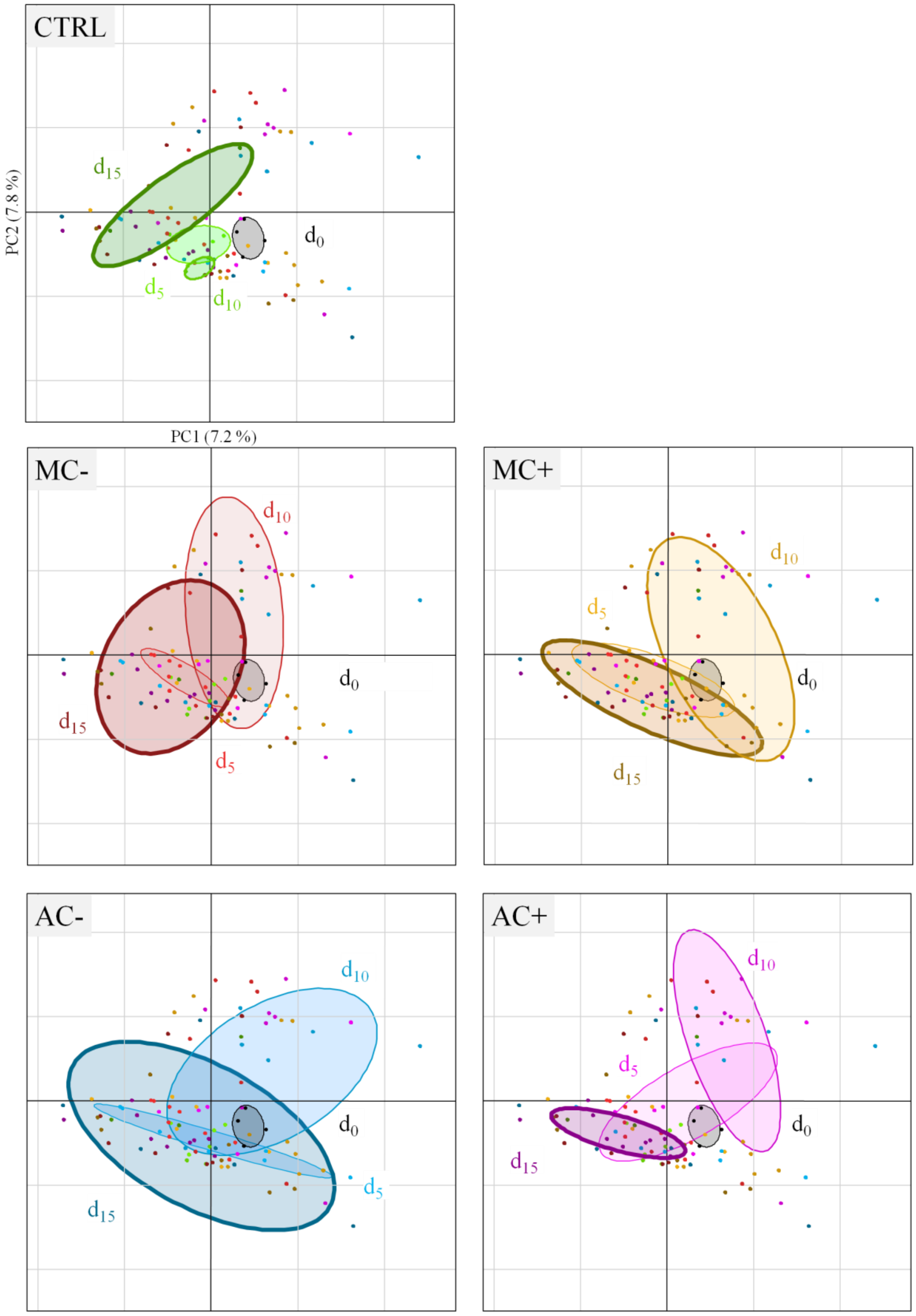

Figure S3 – Principal Component Analysis (PCA) of coral *Astroides calycularis* metabolomic profiles according to treatments (methanolic extracts analyzed in (+)–ESI). MC- – corals in contactless interaction with mimic algae, MC+ – corals in contact interaction with mimic algae, AC- – corals in contactless interaction with algae, AC+ – corals in contact interaction with algae, CTRL – corals alone for controls, at different sampling dates (experimental start at day 0, day 5, day 10 and day 15) (scores plot). PC1 and PC2 represents 7.2 and 7.8 % of variance for a total of 15.0 %. Ellipses are graphical representations of groups without any statistical support.

Table S1 – Coral metabolites determined by UHPLC–(+)–ESIMS (methanolic fraction). RT : Retention time in minutes, BPC Area (%) = base peak chromatograms are given as percentage, mS: mSigma is a constructor quality value of the formula determination. It compares accurate masses and isotopic patterns of experimental formula with theoric ones (value of mSigma closest to zero are representative of maximal fit). Base peak chromatograms were considered for ion formula determination.

Peak	RT (min)	BPC Area (%)	$[M+H]^+$	Ion formula	Annotation	mS
1	0.54	16.8	235.1650	$C_{10}H_{23}N_2O_4$	-	1.2
2	0.57	8.6	387.1775	$C_{18}H_{23}N_6O_4$	dihydrogenated orthidine (?)	3.2
3	0.86	0.6	387.1774	$C_{18}H_{23}N_6O_4$	dihydrogenated orthidine (?)	13.6
4	2.83	20.2	194.0923	$C_9H_{12}N_3O_2$	tubastrine	0.7
5	4.39	2.7	385.1619	$C_{18}H_{21}N_6O_4$	orthidine	4.9
6	4.69	2.1	385.1617	$C_{18}H_{21}N_6O_4$	orthidine	10.0
7	4.83	3.0	385.1619	$C_{18}H_{21}N_6O_4$	orthidine	4.9
8	4.94	2.0	385.1618	$C_{18}H_{21}N_6O_4$	orthidine	7.2
9	5.50	1.4	537.2716	$C_{30}H_{33}N_8O_2$	-	13.2
10	5.83	2.1	537.2718	$C_{30}H_{34}N_8O_2$	-	7.2
11	6.04	2.8	255.1235	$C_{14}H_{15}N_4O$	aplysinopsin	3.6
12	6.12	19.1	269.1390	$C_{15}H_{17}N_4O$	*N*–methylaplysinopsin	2.7
13	6.48	0.2	212.1755	$C_{11}H_{22}N_3O$	-	4.0
14	6.84	2.0	333.0339	$C_{14}H_{14}BrN_4O$	6–bromoaplysinopsin	3.9
15	6.91	10.4	347.0495	$C_{15}H_{16}BrN_4O$	6–bromo–*N*–methylaplysinopsin	18.3
16	7.09	0.9	311.1498	$C_{17}H_{19}N_4O_2$	*N*–propionylaplysinopsin	14.1
17	7.14	0.4	325.1654	$C_{18}H_{21}N_4O_2$	*N*–methyl–*N*–propionylaplysinopsin	19.5
18	7.34	0.5	524.2406	$C_{29}H_{30}N_7O_3$	-	9.3
19	8.62	0.4	198.1848	-	-	-
20	8.84	2.9	214.0892	-	-	-
21	8.90	0.4	249.2058	-	-	-
22	10.87	0.3	440.4097	$C_{27}H_{54}NO_3$	-	3.9

Table S2 – ***Asparagopsis taxiformis* ions responsible for group 2 difference in the Principal Component Analysis (Fig. 6 in main manuscript).** RT: Retention time in minutes, mS: mSigma is a constructor quality value of the formula determination. It compares accurate masses and isotopic patterns of experimental formula with theoric ones (value of mSigma closest to zero are representative of maximal fit). Base peaks chromatograms were considered for ion formula determination.

	positive mode				negative mode			
RT (min)	$[M+H]^+$	Ion formula	Error (ppm)	mS	$[M-H]^-$	Ion formula	Error (ppm)	mS
3.3	-	-	-	-	186.1137	$C_9H_{16}NO_3$	-0.9	3.7
3.9	-	-	-	-	168.1033	$C_9H_{14}NO_2$	-1.7	7.1
4.0	-	-	-	-	214.1454	$C_{11}H_{20}NO_3$	-2.6	5.1
5.8	288.2895	$C_{17}H_{38}NO_2$	0.7	6.1	-	-	-	-
7.3	318.2401	$C_{16}H_{28}N_7$	0	12.3	-	-	-	-
7.8	320.2562	$C_{16}H_{30}N_7$	-1.2	1.2	-	-	-	-
8.0	280.2635	$C_{18}H_{34}NO$	-0.1	7.1	-	-	-	-
8.2	228.2321	$C_{14}H_{30}NO$	0.3	3.2	-	-	-	-
9.7	256.2634	$C_{16}H_{34}NO$	0.5	6.8	-	-	-	-
9.8	280.2631	$C_{18}H_{34}NO$	1.5	8.4	-	-	-	-
10.5	282.2790	$C_{18}H_{36}NO$	0.4	6.2	-	-	-	-
10.9	270.2793	$C_{17}H_{36}NO$	-0.8	2.4	-	-	-	-
12.6	284.2945	$C_{18}H_{38}NO$	1.0	11.4	-	-	-	-
